# Glutathione metabolism as a key regulator of oxidative hippocampal injury in sepsis-associated encephalopathy: an integrated proteomics and metabolomics study

**DOI:** 10.3389/fnins.2025.1671955

**Published:** 2026-01-13

**Authors:** Yanning Li, Linan Wang, Teng Ma, Tao Peng, Lijuan Wang, Junyan Wang, Yunhong Li, Yin Wang

**Affiliations:** 1 School of Basic Medicine, Ningxia Medical University, Yinchuan, Ningxia, China; 2 The First Clinical Medical College of Ningxia Medical University, Yinchuan, Ningxia, China; 3 Science and Technology Center of Ningxia Medical University, Yinchuan, Ningxia, China

**Keywords:** glutathione metabolism, hippocampal injury, multi-omics, oxidative stress, sepsis-associated encephalopathy

## Abstract

**Introduction:**

Sepsis-associated encephalopathy (SAE) is characterized by acute neurological dysfunction and hippocampal damage, with oxidative stress being a key driver of neuronal injury. However, the role of dysfunctional glutathione (GSH) metabolism in hippocampal injury during SAE remains unclear. This study aimed to clarify the molecular and biochemical changes in the hippocampus induced by SAE through multi-omics integration (proteomics and metabolomics), thereby providing a theoretical basis for improved neuroprotective strategies.

**Methods:**

A murine SAE model was established via cecal ligation and puncture (CLP). Subsequent analyses included assessments of hippocampal tissue damage, microglial activation, and cognitive function in mice. Levels of pro-inflammatory cytokines, reactive oxygen species (ROS), and malondialdehyde (MDA) (oxidative stress markers) were detected. Proteomic analysis was performed to identify differentially expressed proteins (DEPs), while metabolomic profiling was used to characterize metabolic changes. Multi-omics integration was conducted to reveal core regulatory networks, and mechanistic validation focused on the expression of Nrf2, HO-1, and GPX4.

**Results:**

The CLP-induced SAE model showed significant hippocampal damage, microglial activation, cognitive deficits, and increased levels of pro-inflammatory cytokines, ROS, and MDA. Proteomic analysis identified 156 DEPs, with glutathione metabolism being the most severely disrupted pathway. Metabolomic results confirmed systemic glutathione depletion and mitochondrial dysfunction, as evidenced by reduced levels of S-lactoylglutathione, carnitine species, and NAD+ intermediates. Multi-omics integration revealed a “high-inflammation, high-oxidation, low-metabolism” triad, which is mainly regulated by the Stat1-(2-carboxypropyl)-Cysteamine-C3 interaction axis. Mechanistic validation further confirmed downregulated expression of Nrf2, HO-1, and GPX4 in CLP mice, establishing a direct link between glutathione dysregulation and neuronal apoptosis.

**Discussion:**

Our findings demonstrate that glutathione metabolism serves as a pivotal hub in the pathogenesis of SAE. The identified glutathione-related pathways provide potential therapeutic targets for alleviating oxidative stress-induced hippocampal injury in SAE, offering new insights for the clinical management of SAE-related neurological damage.

## 1 Introduction

Sepsis-associated encephalopathy (SAE) represents a severe complication of sepsis, defined by diffuse brain dysfunction without direct central nervous system (CNS) infection (Chaudhry and Duggal, 2014). Clinically, SAE presents with cognitive impairment, delirium and even coma, affecting 50%–70% of patients with sepsis. Notably, SAE is associated with a poor prognosis; the mid-late stage mortality rate exceeds 20% (Mazeraud et al., 2020; Young et al., 1990) and the overall mortality rate remains high at 25%–50%, despite advances in critical care. Furthermore, SAE remains notably underdiagnosed and undertreated, and survivors often develop persistent long-term neurocognitive sequelae, including hippocampus-dependent memory impairment, psychiatric disorders and functional disability, which severely impact quality of life (Pan et al., 2022). Pathologically, SAE is characterized by blood-brain barrier disruption, excessive neuroinflammation, and heightened oxidative stress, with the hippocampus consistently emerging as a key injury locus (Gao and Hernandes, 2021; Hoshino, 2025). However, the molecular mechanisms linking systemic inflammation to hippocampal oxidative damage in SAE remain unclear, hindering targeted therapies.

Oxidative stress is central to SAE pathogenesis, driven by an imbalance between excessive reactive oxygen species (ROS) production and impaired antioxidant defense systems (Bozza et al., 2013). Accumulating evidence indicates that sepsis-induced neuroinflammation activates microglia, triggering a pro-inflammatory “cytokine storm” (TNF-α, IL-1β, IL-6) that exacerbates mitochondrial dysfunction and ROS burst in hippocampal neurons (Michels et al., 2015; Yan et al., 2022). Clinical studies note reduced activity of key antioxidant enzymes, such as glutathione (GSH) peroxidase (GPX) in SAE patients, highlighting the potential vulnerability of antioxidant systems in disease progression (Graf et al., 1998). However, the specific metabolic pathways that mediate oxidative hippocampal injury in SAE remain poorly characterized.

The antioxidant defense system, especially GSH metabolism, is linked to neurodegenerative diseases (Wu et al., 2004), but its role in SAE in incompletely defined. As the most abundant intracellular thiol antioxidant, GSH is the primary oxidative stress defense of the brain, coordinating synthesis, recycling, and redox signaling to maintain redox homestasis (Schulz et al., 2000). Within the hippocampus, redox balance is regulated by the glutathione peroxidase/glutathione reductase (GPx/GR) system, which is vnlnerable to systemic inflammation (Mariani et al., 2005), Emerging evidence underscores a “double-hit” mechanism, simultaneous ROS overproduction, and antioxidant depletion that amplifies inflammation and neuronal damage (Gu et al., 2021). Nevertheless, the molecular cascades bridging GSH metabolic dysfunction to neuronal impairment remain poorly elucidated.

Proteomics and metabolomics enable systematic profiling of protein expression and metabolic signatures. Their integration offers synergistic advantages: proteomics uncovers dynamic changes in enzyme abundance, post-translational modifications and signaling pathways, wheras metabolomics captures real-time metabolic flux, substrate-product relationships, and metabolic bottlenecks (Kreitmaier et al., 2023). This integrative multi-omics approach is uniquely powerful for dissecting the pathophysiological mechanisms of complex diseases such as SAE.

This study aimed to elucidate the mechanisms underlying oxidative hippocampal injury in mid-late SAE using an integrated multi-omics strategy. Using a cecal ligation and puncture (CLP) murine model, in vitro microglia-neuron co-cultures, data-independent acquisition (DIA) proteomics and liquid chromatography-mass spectrometry (LC-MS) metabolomics, SAE neuropathology and phenotypes were characterized. Dysregulated metabolic pathways were identified. Multi-omics integration and validation indicated that dysfunctional GSH metabolism may link systemic inflammation to hippocampal injury, highlighting therapeutic potential and advancing the understanding of SAE.

## 2 Materials and methods

### 2.1 Experimental animals

Male C57BL/6J mice [specific-pathogen-free (SPF) grade], aged 8–10 weeks and weighing 20–25 g, were obtained from the Laboratory Animal Center of Ningxia Medical University. The mice were housed in the Animal Experimental Center of the Key Laboratory of Cranial Diseases under SPF conditions, with controlled environmental parameters (temperature: 23–25°C; humidity: 50%–60%) and a 12 h light/dark cycle. Food and water were provided ad libitum. All experimental procedures were approved by the Animal Ethics Committee of Ningxia Medical University (Approval No. 2025-4471) and conducted in strict accordance with Chinese regulations for the ethical use of laboratory animals.

### 2.2 Cecal ligation puncture in mice

The mice were randomly assigned to either the Sham group or the CLP group. Model establishment followed the reported protocol (Rittirsch et al., 2009), mice were anesthetized with isoflurane (3% for induction, 1.0% for maintenance), followed by a midline abdominal incision to expose the cecum, which was ligated distal to the ileocecal valve using 4-0 silk suture and punctured twice with a 21-gauge needle to extrude a small amount of fecal material before being returned to the abdominal cavity for layered closure, while Sham group mice underwent identical procedures without cecal ligation or puncture. Postoperatively, all animals were maintained on a 37°C heating pad until full recovery of righting reflexes postoperatively.

### 2.3 Open field test (OFT)

The OFT was conducted to assess anxiety-like behavior, exploratory activity, and locomotor function in mice. This experiment was performed on day 6 after CLP surgery. Each mouse was gently placed in the center of a clear acrylic open-field arena (40 × 40 × 40 cm). Behavior was recorded for 10 min using an automated tracking system to quantify: (Chaudhry and Duggal, 2014) number of central zone entries (defined as >50% body length crossing the border) and (Young et al., 1990) total ambulatory distance. The apparatus was thoroughly cleaned with 70% ethanol and air-dried between trials to eliminate residual odor cues.

### 2.4 Y-maze

Spatial working memory was evaluated using a symmetrical acrylic Y-maze (This experiment was performed on day 7 after CLP surgery) consisting of three arms (35 cm in length, 5 cm in width, 15 cm in height) positioned at 120° angles to each other. During the 5-min training phase, mice were allowed to freely explore two arms (designated as the start arm and familiar arm), with the third arm (novel arm) blocked by a removable barrier. After a 2-h inter-trial interval, the test phase commenced by unblocking the novel arm. Tracking software quantified number of entries into the novel arm during the 5-min test phase. The maze was cleaned with 70% ethanol between subjects to prevent olfactory interference.

### 2.5 Nissl staining

In postoperative day 7, mice were transcardially perfused with ice-cold phosphate-buffered saline (PBS, pH 7.4) followed by 4% paraformaldehyde (PFA; Biosharp) according to standard protocols. Brain tissues were post-fixed, dehydrated through graded ethanol series, cleared in xylene, and embedded in paraffin. Coronal sections (4 μm thickness) were obtained using a rotary microtome and baked at 60°C for 2 h to enhance tissue adhesion. For Nissl staining, sections were sequentially deparaffinized in xylene (twice, 10 min each), rehydrated through a graded ethanol series (100, 95, 90%, 85, 80, and 70%; 2 min each), and stained with cresyl violet acetate solution (Solarbio, at 60°C for 30 min in light-protected conditions). After rinsing with distilled water, sections were differentiated in 70% ethanol under microscopic monitoring until optimal contrast was achieved. Rapid dehydration was performed using graded ethanol (95% and 100%, 30 s each), followed by xylene clearance (twice, 5 min each). Sections were coverslipped with neutral balsam (Beyotime) and air-dried. Digital images were acquired using a high-resolution slide scanner at 40 × magnification. All experimental groups were processed simultaneously to ensure staining consistency. Quantitative analysis was performed using ImageJ software by counting the number of Nissl-positive neurons in the hippocampal CA1, CA3, and dentate gyrus (DG) regions.

### 2.6 Brain electrodes

Mice were anesthetized with isoflurane. The head was securely fixed in a stereotactic frame to ensure bilateral symmetry and alignment with bregma. After disinfecting the scalp with iodophor solution, a midline incision was made to expose the skull, gently removing the periosteum and associated connective tissue to expose the anterior and posterior fontanelles and the Bregma point. Subsequently, according to the method described in the previous study (Liu et al., 2020), electrodes were implanted into the hippocampus using a stereotactic device. The stereotactic coordinates of the hippocampus were set as X = 1.5 mm, Y = 2.0 mm, and Z = 2.0 mm with bregma as the origin. Electrodes were implanted one day prior to surgery. Then measured the brain electrodes in mice by Taimeng BL420N Biological Information Signal Acquisition System, with preliminary measurements performed on the first 2 days post-surgery and formal determination carried out on the third day post-surgery.

### 2.7 Immunofluorescence (IF)

After transcardial perfusion in mice, brain tissues were harvested and pretreated with gradient dehydration in 20% and 30% sucrose solutions at 4°C (to reduce ice crystal damage), then embedded in optimal cutting temperature compound and sectioned for 20 μm thickness using cryostat. For IF staining, fixed with 4% PFA for 15 min, permeabilized with 0.3% Triton X-100 in PBS for 10 min, and blocked with 5% normal donkey serum for 1 h at room temperature (RT). Sections were then incubated overnight at 4°C with primary antibodies against Iba1 (1:300, Proteintech), diluted in blocking buffer. After washing with PBS (3 × 5 min), sections were incubated with species-matched Alexa Fluor-conjugated secondary antibodies (1:500, Invitrogen) for 2 h at RT protected from light. Finally, sections were mounted with anti-fluorescence quenching mounting medium (with DAPI) (Solarbio) and imaged using microscope with consistent laser power and gain settings across samples. Quantification of Iba1^+^ cells was performed using ImageJ software, with results expressed as fluorescence intensity per mm^2^ or mean fluorescence intensity (MFI).

### 2.8 Proteomic and metabolomic analyses

#### 2.8.1 Sample preparation

In postoperative day 7, hippocampal tissues were dissected on ice using sterile instruments (*n* = 6 for each groups), rinsed with 0.9% saline, and placed into pre-chilled cryotubes for immediate liquid nitrogen freezing. The samples were stored at −80°C until being packed in dry ice and sent to Maiwei Metabolomics (Wuhan, China) for analysis.

#### 2.8.2 Quantitative proteomic analysis

Following enzymatic digestion and desalting, 100 μg of protein samples were subjected to LC-MS/MS analysis. Raw data were analyzed and normalized via DIA-NN v1.8.1. Proteins with a fold change (FC) ≥ 1.5 or FC ≤ 0.6667, coupled with a *P*-value ≤ 0.05, were defined as significantly differential proteins (DEPs). Subsequent bioinformatics analyses of DEPs included Gene Ontology (GO) functional annotation and enrichment, Kyoto Encyclopedia of Genes and Genomes (KEGG) pathway enrichment analysis^1^, and protein-protein interaction (PPI) analysis via the STRING database.^2^

#### 2.8.3 Widely targeted metabolomics analysis

Frozen tissue specimens were homogenized and extracted using ice-cold methanol:water (7:3, v/v) supplemented with isotope-labeled internal standards,followed by multi-platform LC-MS/MS analysis. After preprocessing and quality control analysis of the raw data, differential metabolites (DEMs) analysis were identified by variable importance in the projection (VIP) > 1 and *P*-value < 0.05).

#### 2.8.4 Enzyme-linked immunosorbent assay (ELISA)

Serum were collected from all experimental groups after mice were sacrificed and analyzed for inflammatory cytokine levels (TNF-α and IL-1β) using commercial ELISA kits (Jingmei, Jiangsu). Briefly, after preparing serial dilutions of the standards, 50 μL of standards or 10 μL of serum (mixed with 40 μL diluent) were added to the appropriate wells of the microplate, with blank wells containing assay buffer only. Following incubation at 37°C for 30 min and five washes with washing buffer, 50 μL of horseradish peroxidase(HRP)-conjugated detection antibody was added to each well (except blanks) and incubated for an additional 30 min at 37°C. After repeating the washing procedure, the chromogenic reaction was initiated by adding 50 μL each of TMB substrates A and B, followed by 10 min of dark incubation at 37°C. The reaction was terminated with 50 μL of stop solution, and the absorbance was measured at 450 nm using a microplate reader (BioTek Eon). All samples were assayed in duplicate, with standard curves generating *R*^2^ values > 0.99 and inter-assay coefficients of variation < 10% ensuring assay reliability.

#### 2.8.5 Cell culture

BV2 microglial cells and HT22 hippocampal neurons (institutional cell bank) were routinely maintained in high-glucose DMEM (Gibco) supplemented with 10% fetal bovine serum (FBS, BI) and 1% penicillin/streptomycin (Biosharp) at 37°C in a 5% CO_2_ humidified atmosphere. For revival, cryopreserved vials were rapidly thawed in a 37°C water bath, the cell suspension was transferred to complete medium followed by centrifugation (1,000 rpm, 5 min). The pellet was resuspended and seeded into T25 flasks (Corning), with medium replacement every 1–2 days until 90% confluence was achieved. For subculture, cells were washed with PBS, detached using 0.25% trypsin-EDTA (Solarbio) at 37°C for 3 min, and digestion was terminated with double volume of complete medium. After centrifugation (1,000 rpm, 5 min), cells were resuspended and either passaged for passaging(1:3–1:5) or cryopreserved in freezing medium (90% FBS and 10% DMSO) using a programmed freezing container at −80°C overnight before long-term storage in liquid nitrogen.

To establish the neuroinflammatory model in vitro, BV2 cells at 80%–90% confluence were stimulated with 1 μg/mL ultrapure LPS (lipoplysaccharide, Sigma) for 24 h. The conditioned medium (CM) was then collected, centrifuged (3,000 rpm, 10 min), filtered through 0.22 μm membranes (Millipore), and applied to HT22 neurons for 12 h treatment, while control groups received fresh complete medium.

#### 2.8.6 Mitochondrial membrane potential detection

Mitochondrial membrane potential was evaluated using the fluorescent cationic dye JC-1 (5,5’,6,6’-tetrachloro-1,1’,3,3’-tetraethylbenzimidazolcarbocyanine iodide; KGI Biotechnology, China) according to the manufacturer’s protocol with modifications. Briefly, 1 × 10^5^–10^6^ cells were harvested, washed twice with ice-cold PBS (1,000 rpm, 5 min, 4°C), and resuspended in 500 μL pre-warmed JC-1 working solution (1 × incubation buffer). After incubation at 37°C in 5% CO_2_ for 15–20 min, cells were washed twice with 1 × incubation buffer (1,000 rpm, 5 min, RT), resuspended in 500 μL fresh buffer, and immediately analyzed by flow cytometry (BD Accuri C6). All procedures were performed under light-protected conditions, and data acquisition was completed within 30 min post-staining to ensure signal stability, and the results were processed using FlowJo 10.1 software.

#### 2.8.7 ROS

Intracellular ROS levels were quantified using the fluorescent probe 2’,7’-dichlorodihydrofluorescein diacetate (DCFH-DA) from a commercial ROS Assay Kit (KGI Biotechnology, China). Briefly, cells were adjusted to a density of 1 × 10^6^ cells/mL in PBS, and incubated with 10 μM DCFH-DA working solution (prepared by 1:1000 dilution of 10 mM stock) at 37°C for 20 min in the dark, with gentle inversion every 3–5 min to ensure uniform probe loading. After centrifugation (1,000 rpm, 3 min, RT), cells were washed three times with serum-free medium to remove extracellular probe, and finally resuspended in phenol red-free medium for immediate analysis. Fluorescence intensity was measured by flow cytometry within 30 min, and unstained cells for background subtraction. Data were analyzed using FlowJo 10.1 software.

### 2.9 GSH and malondialdehyde (MDA)

The GSH and MDA contents in hippocampal tissue were detected by using the kit provided by Nanjing Jiancheng Institute of Biological Engineering, respectively, and the operation was carried out according to the steps in the instructions, wherein, the calculation formula is as follows:


$$M\,D\,A\,\left(n\,m\,o\,l/m\,g\,p\,r\,o\,t\right)=\frac{A\,M\,e\,a\,s\,u\,r\,e-A\,B\,l\,a\,n\,k}{A\,S\,t\,a\,n\,d\,a\,r\,d-A\,B\,l\,a\,n\,k}\times C\,s\,t\,a\,n\,d\,a\,r\,d÷C\,p\,r$$

*C*_*standard*_: standard concentration, 10 nmol/mL;

*Cpr*: sample protein concentration, mgprot/mL, measured by BCA.


$$G\,S\,H\,\left(u\,m\,o\,l/g\,p\,r\,o\,t\right)=\frac{A\,M\,e\,a\,s\,u\,r\,e-A\,B\,l\,a\,n\,k}{A\,S\,t\,a\,n\,d\,a\,r\,d-A\,B\,l\,a\,n\,k}\times C\,s\,t\,a\,n\,d\,a\,r\,d\times 2÷C\,p\,r$$

*C*_*standard*_: standard concentration, 20 μmol/L;

2: Sample pretreatment dilution factor;

*Cpr*: sample protein concentration, *mgprot/mL*.

### 2.10 Western blot

Hippocampal tissues were homogenized in lysis buffer (KGI Biotechnology), centrifuged at 12,000 rpm (4°C, 15 min) to collect the supernatant. Protein concentration was determined using a BCA assay kit (KGI Biotechnology), and equal amounts of protein (30 μg per lane) were mixed with 5 × Loading buffer, denatured at 95°C for 5 min, and separated on 10% SDS-PAGE gels (80 V for 30 min, then 120 V for 50 min). Proteins were transferred to PVDF membranes (Millipore) at 300 mA for 45 min in ice-cold transfer buffer. Membranes were blocked with 5% non-fat milk in TBST (0.5% Tween-20 in Tris-Buffered Saline) for 1 h at room temperature, followed by incubation with primary antibodies (1:1000) overnight at 4°C. After washing with TBST HRP-conjugated secondary antibodies (1:5000) were applied (1 h, RT). Protein bands were visualized using enhanced chemiluminescence substrate (Thermo Fisher Scientific) and imaged with a ChemiDoc system (Bio-Rad), then quantified via ImageJ.

### 2.11 Statistical analysis

All data were analyzed using GraphPad Prism 9.5 (GraphPad Software, San Diego, CA, USA). Quantitative data are presented as the mean ± standard error of the mean (SEM). Comparisons between two groups were performed using the independent samples *t*-test. A *P*-value < 0.05 (*P < 0.05, ^**^P < 0.01, ^***^P < 0.001, ^****^P < 0.0001) was considered statistically significant.

## 3 Results

### 3.1 Neuropathological and behavioral manifestations of hippocampal damage in SAE

**FIGURE 1:**
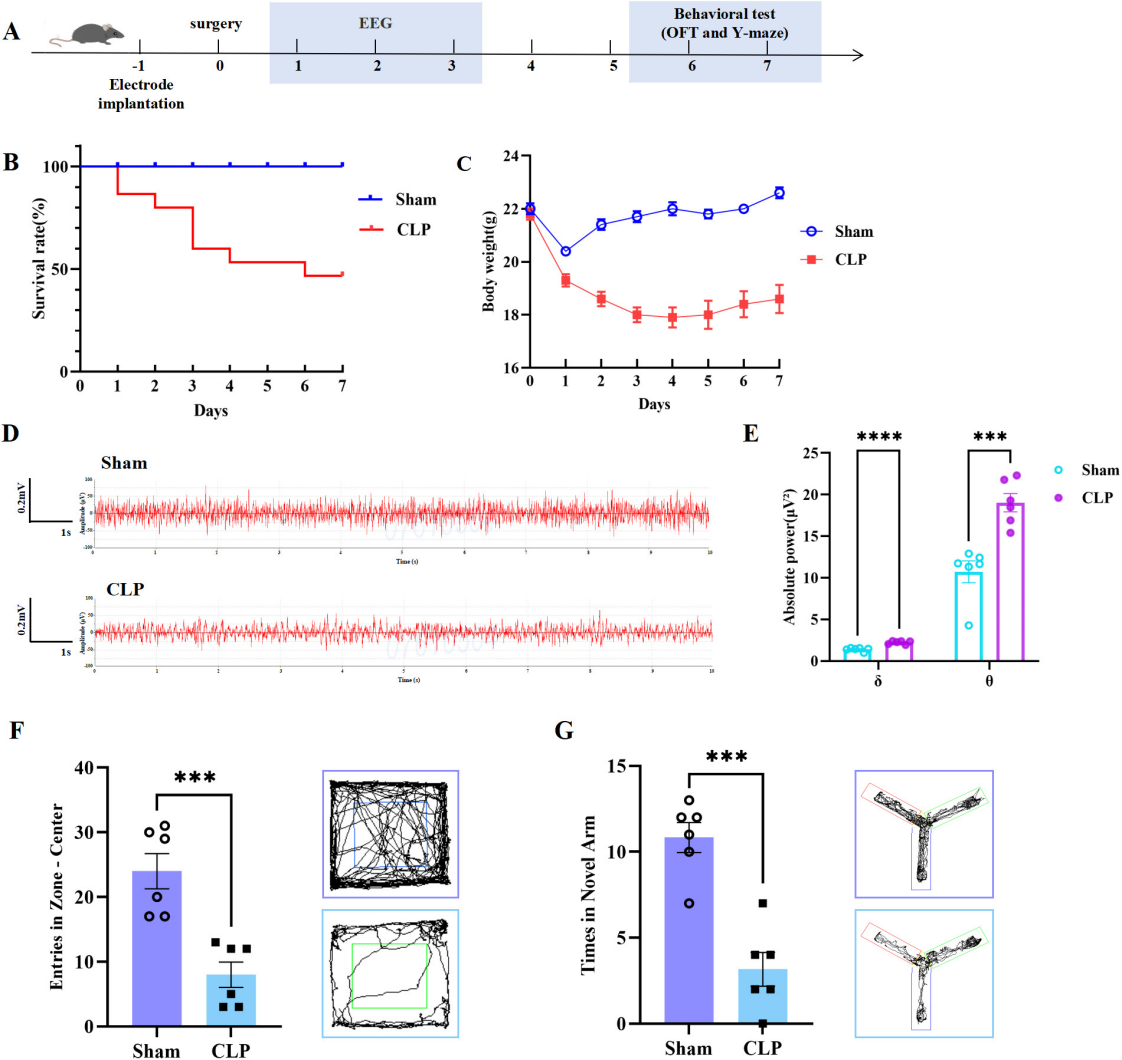
Multidimensional characterization of SAE. **(A)** Experimental procedure; **(B)** Survial rate of 7 days in mice (*n* = 6 of Sham and *n* = 14 of CLP); **(C)** Body weight of 7 days in mice (*n* = 6 of Sham and *n* = 14 of CLP); **(D)** EEG in mice (*n* = 3); **(E)** Absolute power about δ and θ waves in two groups (*n* = 3); **(F)** Entries into the central zone (Left) and movement path (Right. Upper panel: Sham group; Lower panel: CLP group) by OFT (*n* = 6); **(G)** Times in Novel arm (Left) and exploration trajectory (Right. Upper panel: Sham group; Lower panel: CLP group) by Y-maze (*n* = 6). (^***^: *P* < 0.001; ^****^: *P* < 0.0001). SAE, sepsis-associated encephalopathy; CLP, cecal ligation and puncture; EEG, electroencephalogram; OFT, open field test.

Figure 1A illustrates a schematic of the experimental workflow, detailing the temporal progression of sham and CLP mice during SAE model establishment. Within 7 days post-surgery, the CLP group exhibited a 42% mortality rate, whereas all sham mice survived (100% survival) (Figure 1B). Body weight analysis revealed that CLP mice lost 14.7 ± 6.0% of initial weight by day 7 (initial: 21.8 ± 0.5 g; day 7: 18.6 ± 1.3 g), while sham mice maintained a 2.7 ± 0.24% weight gain (initial: 22 ± 0.5 g; day 7: 22.6 ± 0.5 g) (Figure 1C). Electrophysiologically, CLP mice displayed reduced EEG frequency (Figure 1D) and significantly higher quantities of δ (1–4 Hz) and θ (4–12 Hz) waves compared to the sham group (Figure 1E), consistent with established SAE electrophysiological signatures (Pantzaris et al., 2021). anxiety-like behavioral alterations shown by decreased central zone entries in the open field test (OFT) (Figure 1F); and spatial working memory impairment evidenced by reduced novel arm entries in the Y-maze test (Figure 1G).

**FIGURE 2:**
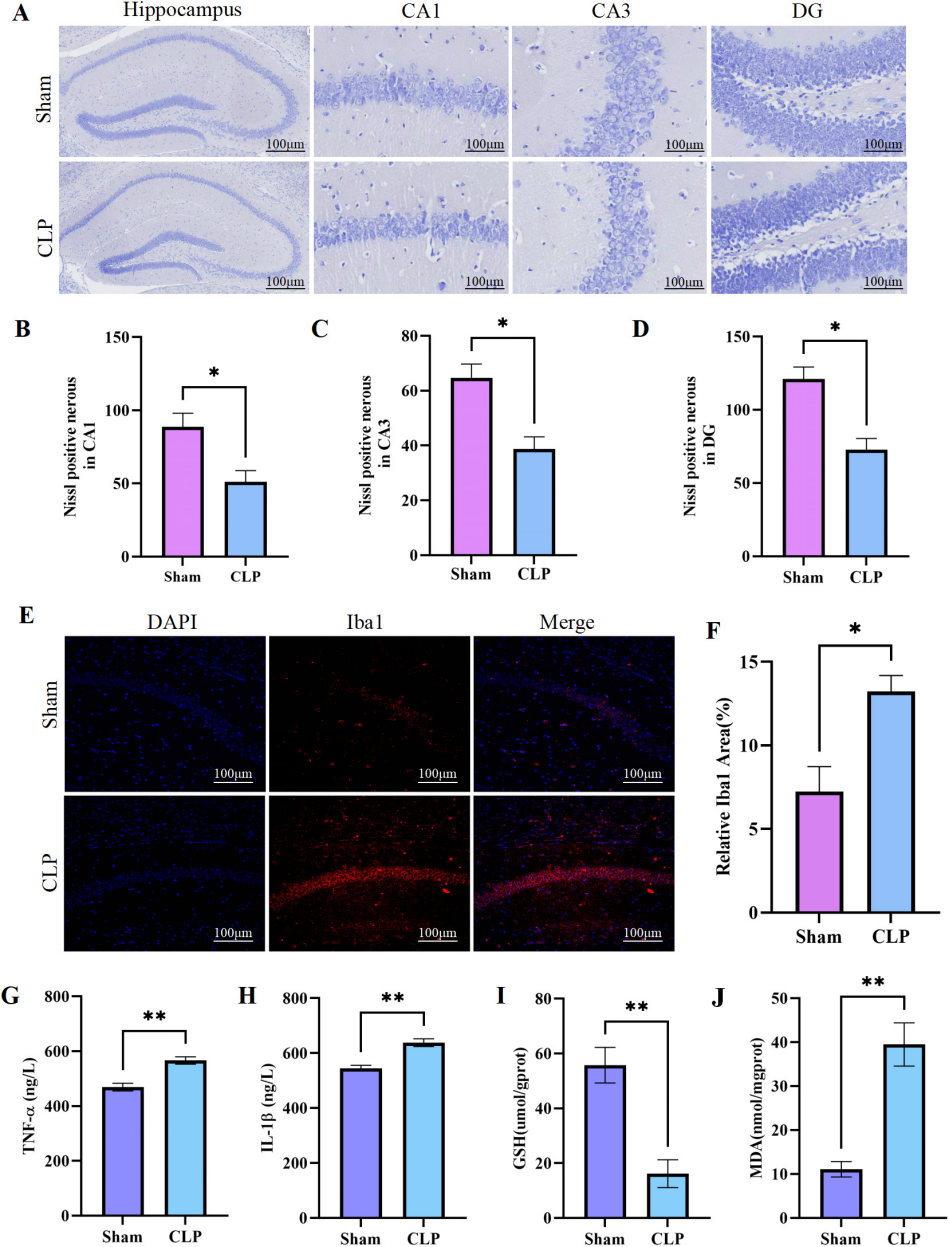
Neuroinflammatory response induced by cecal ligation and puncture (CLP). **(A)** Nissl Staining in hippocampus and different areas; **(B–D)** The counts of Nissl positive nerous in CA1, CA3, and DG; **(E)** Iba1 expression in the CA1 region by immunofluorescence; **(F)** The MFI of Iba1 was measured in the CA1 region; **(G)** Expression of TNF-α by ELISA in serum; **(H)** Expression of IL-1β by ELISA in serum; **(I)** Determination of reduced GSH content in hippocampal tissue; **(J)** Measurement of MDA content in hippocampal tissue (*n* = 3). (*: *P* < 0.05; ^**^: *P* < 0.01). DG, dentate gyrus; IF, immunofluorescence; MFI, mean fluorescence intensity; ELISA, enzyme-linked immunosorbent assay; GSH, glutathione; MDA, malondialdehyde.

The CLP procedure triggered a cascade of systemic inflammatory responses that propagated to the CNS. Histopathological analysis revealed marked neuronal degeneration in CLP mice, with significant reductions in Nissl-positive neuron counts in hippocampal CA1, CA3, and DG subregions (Figures 2A–D). This neuronal damage was accompanied by robust neuroinflammation, as indicated by dramatically increased Iba1-positive microglia (Figures 2E, F). Systemically, CLP mice exhibited significantly elevated serum levels of pro-inflammatory cytokines TNF-α and IL-1β (Figures 2G, H), while hippocampal oxidative stress was enhanced, as shown by depleted GSH levels and accumulated MDA (Figures 2I, J), suggesting synergistic effects of inflammation and oxidative damage on neuronal injury.

In summary, CLP successfully induced SAE with characteristic features: reduced survival and body weight, anxiety-like behavior, cognitive impairment, systemic inflammation, and hippocampal neuronal damage, heightened neuroinflammation and increased oxidative stress. These results recapitulate the core pathological features of clinical SAE, including hippocampal dysfunction and neuroinflammatory responses, providing a reliable platform for mechanistic investigations.

### 3.2 Microglia-derived mediators drive neuronal apoptosis and oxidative stress in SAE

**FIGURE 3:**
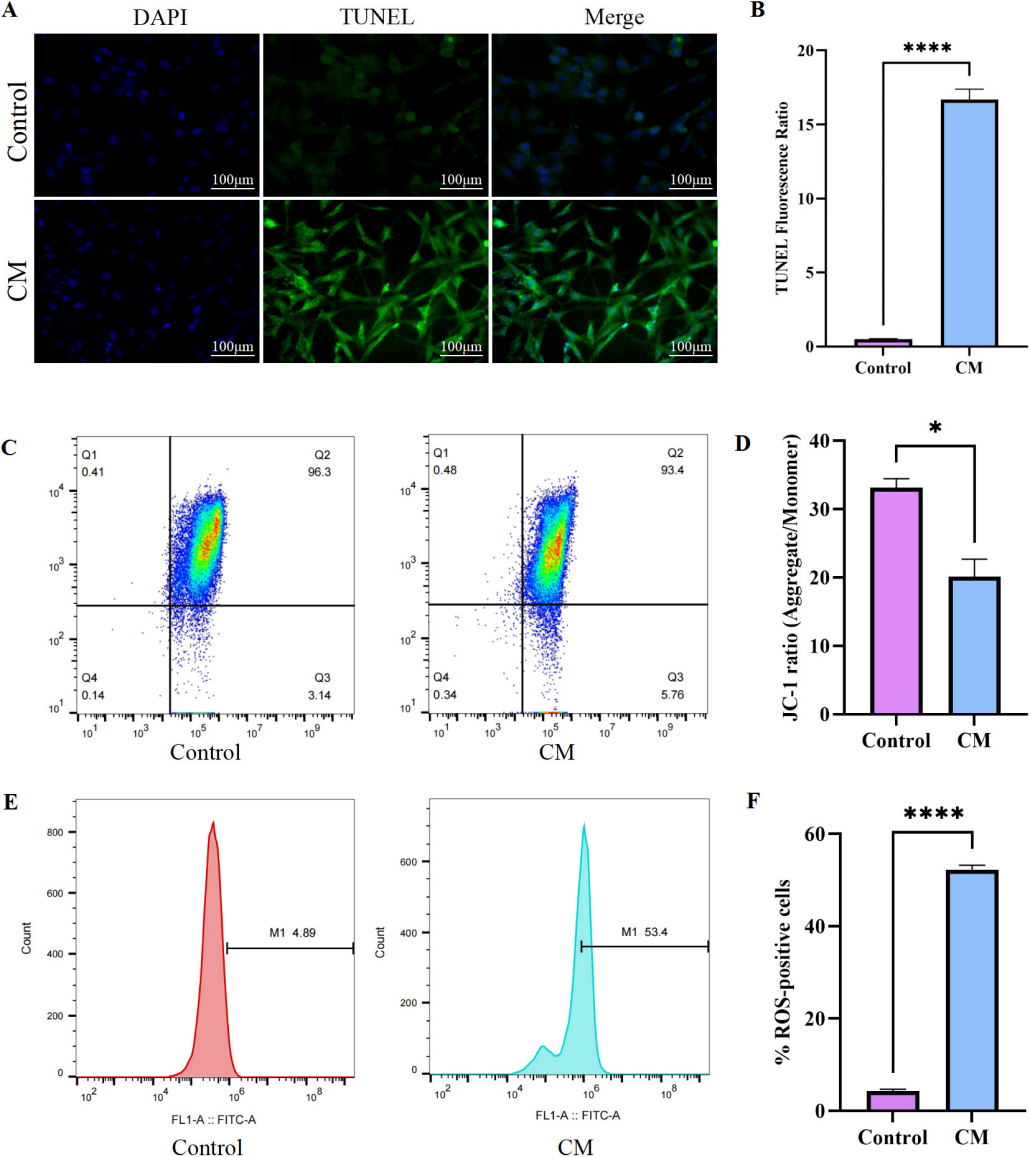
Microglia-induced neuronal oxidative damage and apoptosis *in vitro*. **(A)** Representative images of TUNEL staining in control and CM groups; **(B)** Quantitative analysis of TUNEL-positive cells; **(C)** JC-1 fluorescence staining for mitochondrial membrane potential assessment; **(D)** JC-1 ratio (Aggregate/Monomer); **(E)** Measurement of ROS levels using DCFH-DA fluorescence probe; **(F)** % ROS-positive cells. Experiments were independently repeated three times, with results representing the average of three biological replicates. (*: *P* < 0.05; ^****^: *P* < 0.0001). CM, conditioned medium; JC-1, 5,5’,6,6’-tetrachloro-1,1’,3,3’-tetraethylbenzimidazolcarbocyanine iodide; ROS, reactive oxygen species; DCFH-DA, 2’,7’-dichlorodihydrofluorescein diacetate.

To elucidate microglia-mediated neurotoxicity in SAE, HT22 hippocampal neurons were exposed to CM from LPS-activated BV2 microglia. Analysis of inflammatory cytokines in CM revealed significant upregulation of pro-inflammatory cytokines, including TNF-α and IL-1β (Supplementary Figure 2). Neuronal apoptosis was significantly elevated in CM-treated groups, as evidenced by TUNEL-positive cells (Figures 3A, B). Flow cytometric analysis using JC-1 probe revealed impaired mitochondrial membrane potential (ΔΨm) in CM-treated neurons, characterized by a decreased aggregate/monomer fluorescence ratio (Figures 3C, D), indicating mitochondrial dysfunction. Oxidative stress was robustly induced in CM-treated neurons, as shown by concurrent depletion of intracellular GSH and accumulation of MDA (Supplementary Figure 1), accompanied by robust ROS overproduction, reflected by increased DCFH-DA fluorescence intensity (Figures 3E, F). These findings align with observations *in vivo*, suggesting that the oxidative stress responses in HT22 neurons may be mediated through microglia-derived inflammatory factors. Together, these results demonstrate that microglia-derived inflammatory mediators promote neuronal injury through synergistic mitochondrial impairment and oxidative damage pathways, a critical mechanistic link between neuroinflammation and hippocampal dysfunction in SAE.

### 3.3 Proteomics reveals GSH metabolism as a pivotal pathway in hippocampal tissue

**FIGURE 4:**
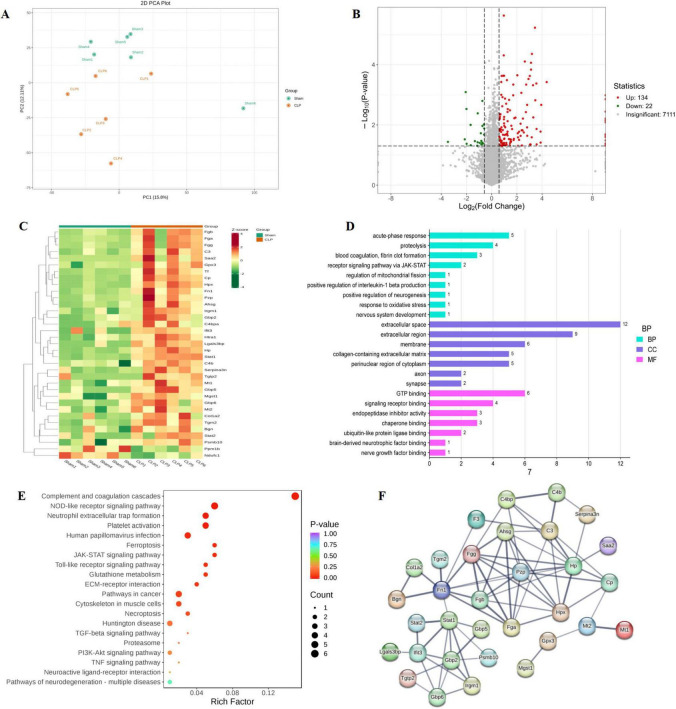
Quantitative proteomics analysis results (*n* = 6). **(A)** (PCA); **(B)** Volcano plot of DEPs; **(C)** Hierarchical clustering heatmap of DEPs in CLP vs. Sham groups; **(D)** GO enrichment analysis of DEPs; **(E)** KEGG pathway enrichment analysis of DEPs; **(F)** PPI network. PCA, principal component analysis; DEPs, differential proteins; GO, Gene Ontology; KEGG, Kyoto Encyclopedia of Genes and Genomes; PPI, protein-protein interaction.

To characterize hippocampal protein perturbations in SAE, DIA-based quantitative proteomics was performed on sham and CLP mice. Principal component analysis (PCA) showed distinct sample clustering, indicating significant proteomic divergence (Figure 4A). A total of 7,267 proteins were identified (Supplementary Table 1), which 156 DEPs were detected, including 134 upregulated and 22 downregulated proteins (Supplementary Table 2), as visualized in the volcano plot (Figure 4B).

Given the central role of oxidative stress in SAE pathogenesis, we focused on 35 oxidative stress-related DEPs. Hierarchical clustering of these 35 DEPs by Z-scores revealed two distinct clusters (Figure 4C): upregulated proteins (red in CLP samples) including ROS-scavenging metallothioneins (Mt1/2), antioxidant enzyme glutathione peroxidase 3 (Gpx3), and oxidative stress-responsive transcription factors Stat1/2; and downregulated proteins (green in CLP samples) such as mitochondrial ROS homeostasis regulator Ppm1b and ROS-exacerbating mitochondrial complex I subunit Ndufc1.

GO enrichment analysis (Figure 4D) linked DEPs to biological processes including oxidative stress response, neurogenesis regulation, acute-phase response, JAK-STAT signaling, and mitochondrial fission, as well as extracellular matrix (ECM) structural constituent and GTP binding. Consistently, KEGG pathway analysis (Figure 4E) highlighted key signaling cascades, including GSH metabolism, ferroptosis, TNF-β signaling pathway, complement and coagulation cascades, NOD-like receptor signaling pathway and pathways of neurodegeneration - multiple disease, indicating crosstalk between oxidative stress, inflammation and fibrotic in SAE pathogenesis.

The PPI network analysis (Figure 4F) identified Stat1 and C3 as central hub nodes, with robust interactions spanning both antioxidant proteins, such as Gpx3 and Mt1/2, and pro-inflammatory mediators including Gbp2, Gbp5, and Gbp6. Collectively, integrated bioinformatics delineated a molecular framework linking oxidative stress, neuroinflammation and ECM dynamics in SAE.

### 3.4 Metabolomics confirms GSH dysregulation across CLP mice

**FIGURE 5:**
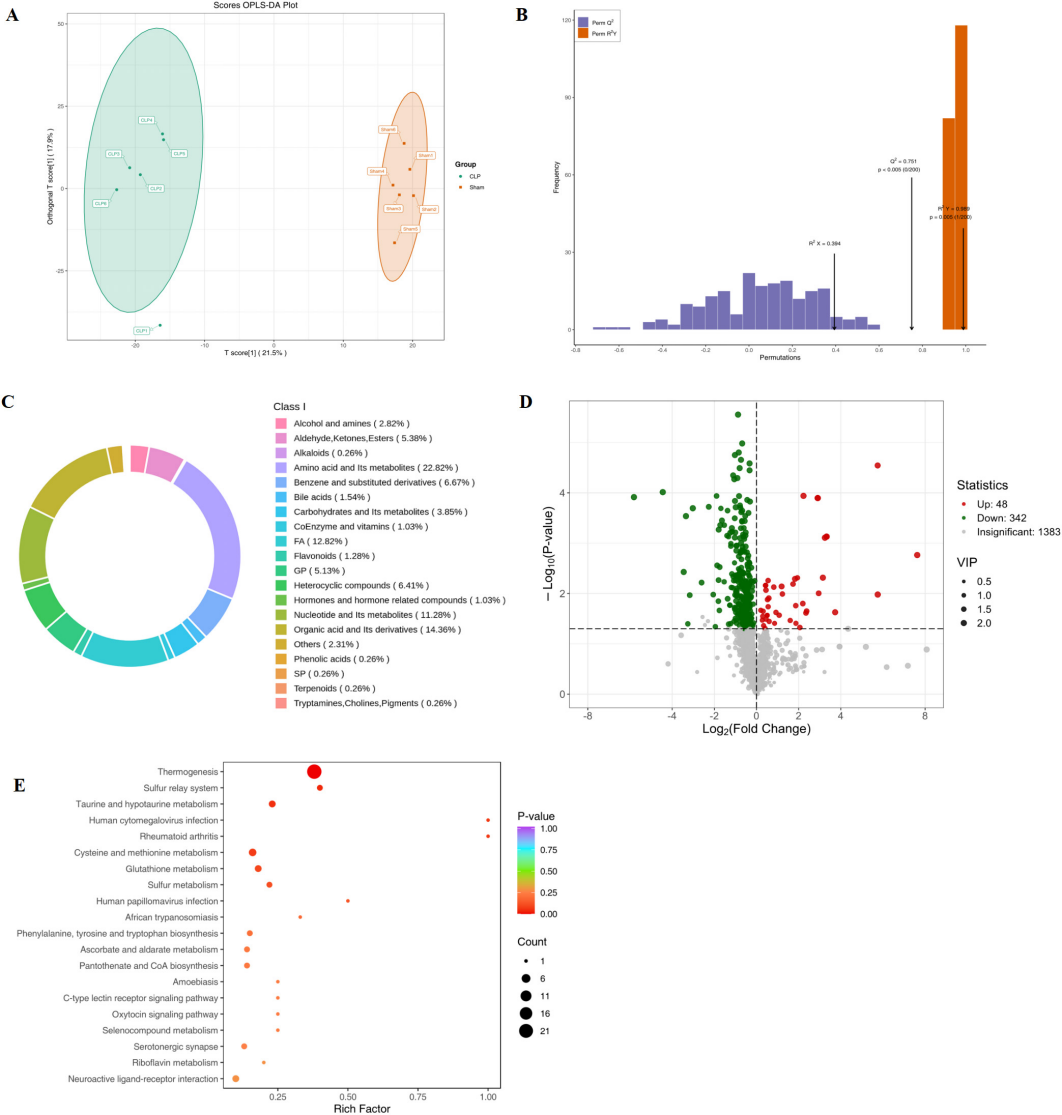
Metabolomics analysis results (*n* = 6). **(A)** OPLS-DA score plot; **(B)** Validation of OPLS-DA model; **(C)** Circular plot of the DGMs categories; **(D)** Volcano plot of the DGMs; **(E)** Enrichment analysis of differential metabolite KEGG. OPLS-DA, orthogonal partial least squares discriminant analysis; DEMs, differential metabolites.

Comprehensive metabolomics profiling of hippocampal tissues from CLP and sham mice was conducted to characterize metabolic perturbations in SAE. Orthogonal partial least squares-discriminant analysis (OPLS-DA) demonstrated clear group separation with robust model reliability (R^2^Y = 0.92) and predictive validity (Q^2^ = 0.85), confirming distinct metabolic signatures (Figures 5A, B). A total of 390 DEMs were identified (Supplementary Table 3). As shown in Figure 5C, the DGMs were composed of different categories, espically in organic acid and its derivatives and nucleotide and its metabolites, with 48 upregulated and 342 downregulated species (Figure 5D). Focusing on oxidative stress-related metabolism (consistent with our proteomic findings), 85 DEMs were annotated to oxidative stress pathways (see Supplementary Table 4). KEGG pathway enrichment analysis (Figure 5E) demonstrated predominant involvement in glutathione metabolism, alongside themogenesis, oxytocin signaling pathways and neuroactive ligand-receptor interaction, reinforcing cross-talk between redox imbalance and neurophysiological dysfunction.

**FIGURE 6:**
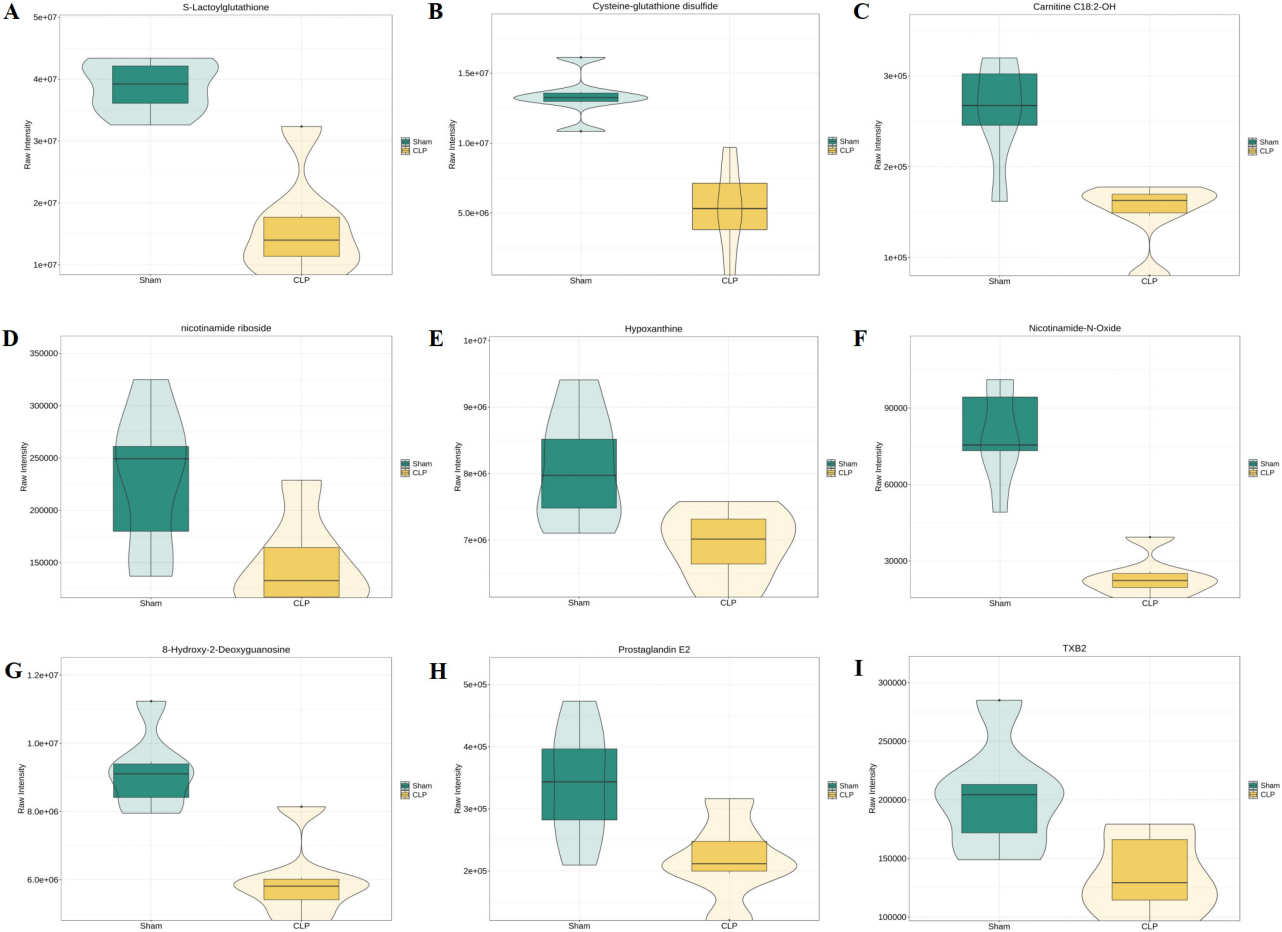
Relative abundance changes of key metabolites in Sham and CLP groups by Violin plots. **(A–I)** Differential abundance of S-Lactoylglutathione, Cysteine-glutathione disulfide, Carnitine C18:2-OH, nicotinamide riboside, Hypoxanthine, nicotinamide-N-Oxide, S-Hydroxy-2-Deoxyguanosine, Prostagtandin E2 and TXB2. TXB2, thromboxane B2.

Key metabolites with significant abundance changes are shown in Figure 6, with GSH system alterations as the central focus. GSH-related metabolites, including S-lactoylglutathione and cysteine-GSH disulfide (Figures 6A, B), were downregulated, indicating compromised antioxidant capacity. Concurrently, depletion of carnitine species (C18:2-OH, Figure 6C) indicated severe mitochondrial dysfunction, while diminished nicotinamide riboside (Figure 6D) potentially exacerbated energy deficits and ROS accumulation in neurons. Other notable changes included decreased hypoxanthine (Figure 6E), suggesting disrupted purine metabolism and impaired ATP regeneration; reduced NAD^+^ biosynthesis intermediates (nicotinamide-N-oxide, Figure 6F); and lowered DNA oxidation marker 8-OHdG (Figure 6G), which may correlate with hippocampal neuronal loss. Inflammatory mediators PGE2 and TXB2 (Figures 6H, I) were downregulated at day 7, differing from acute-phase responses. These findings highlight glutathione system dysregulation as a prominent metabolic feature and potential biomarker of SAE.

### 3.5 Multi-omics integration and mechanistic validation

**FIGURE 7:**
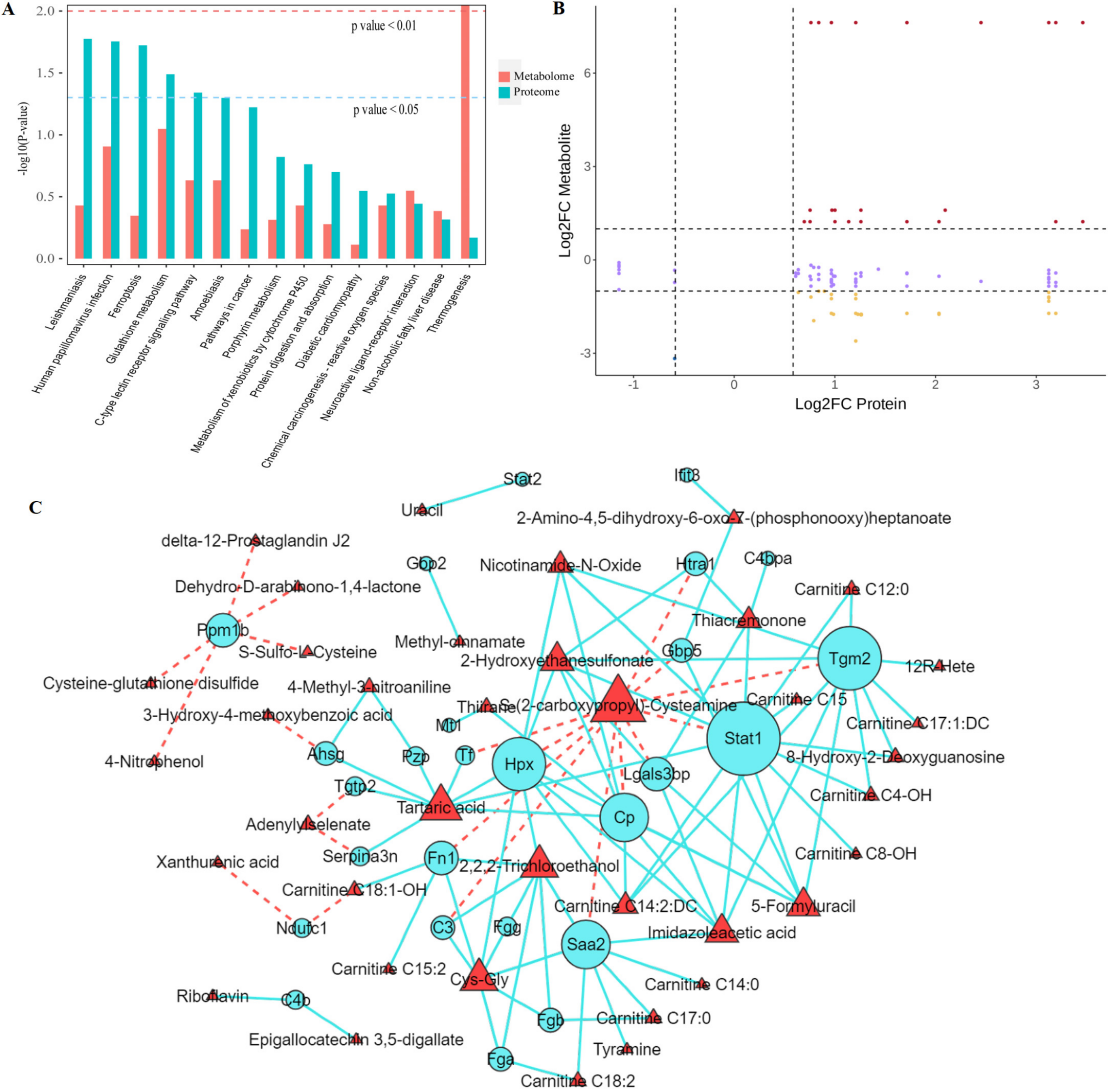
Integrated multiomics analysis results. **(A)** KEGG pathways commonly enriched in proteomic and metabolomic datasets; **(B)** Nine-quadrant plot showing protein-metabolite correlation patterns: consistent differential expression (Quadrants 3, 7), inverse regulatory trends (Quadrants 1, 9), and unidirectional changes (Quadrants 2, 4, 6, 8); **(C)** Molecular interaction network of key proteins and metabolites.

Integrated proteomic and metabolomic analyses of hippocampal tissues delineated a molecular signature characterized by “high inflammation, elevated oxidative stress, and impaired metabolism” in SAE. Three pathways showed significant enrichment (FDR-corrected *P* < 0.01, enrichment factor > 2.0): glutathione metabolism (hsa00480), complement and coagulation cascades (hsa04610), and fatty acid degradation (hsa00071). Multi-omics pathway mapping (Figure 7A) revealed interconnections among glutathione metabolism, ferroptosis, and neuroactive ligand-receptor interaction pathways, showed strong association with mitochondrial function and neuronal damage.

A total of 128 protein-metabolite pairs (Pearson r > 0.8, *p* < 0.05) were visualized in nine-quadrant plots (Figure 7B and Supplementary Table 5), which stratifies the coordinated expression trends (i.e., co-upregulation, co-downregulation, or inverse regulation) of proteins and metabolites to clarify potential regulatory relationships between the two omics layers. To further characterize these interactions, we constructed a molecular interaction network (Figure 7C) revealed three functionally interconnected modules: (Chaudhry and Duggal, 2014) an inflammation cluster centered on Stat1- s (2-carboxypropyl)- Cysteamine- C3 interactions, (Young et al., 1990) an oxidative stress module linking Gpx3 with glutathione metabolites, and (Mazeraud et al., 2020) an energy metabolism network involving Ndufc1 and carnitines species. Collectively, these modules link proteomic and metabolomic dysregulation to the three hallmarks of SAE: neuroinflammation, oxidative damage, and energy metabolism dysfunction.

**FIGURE 8:**
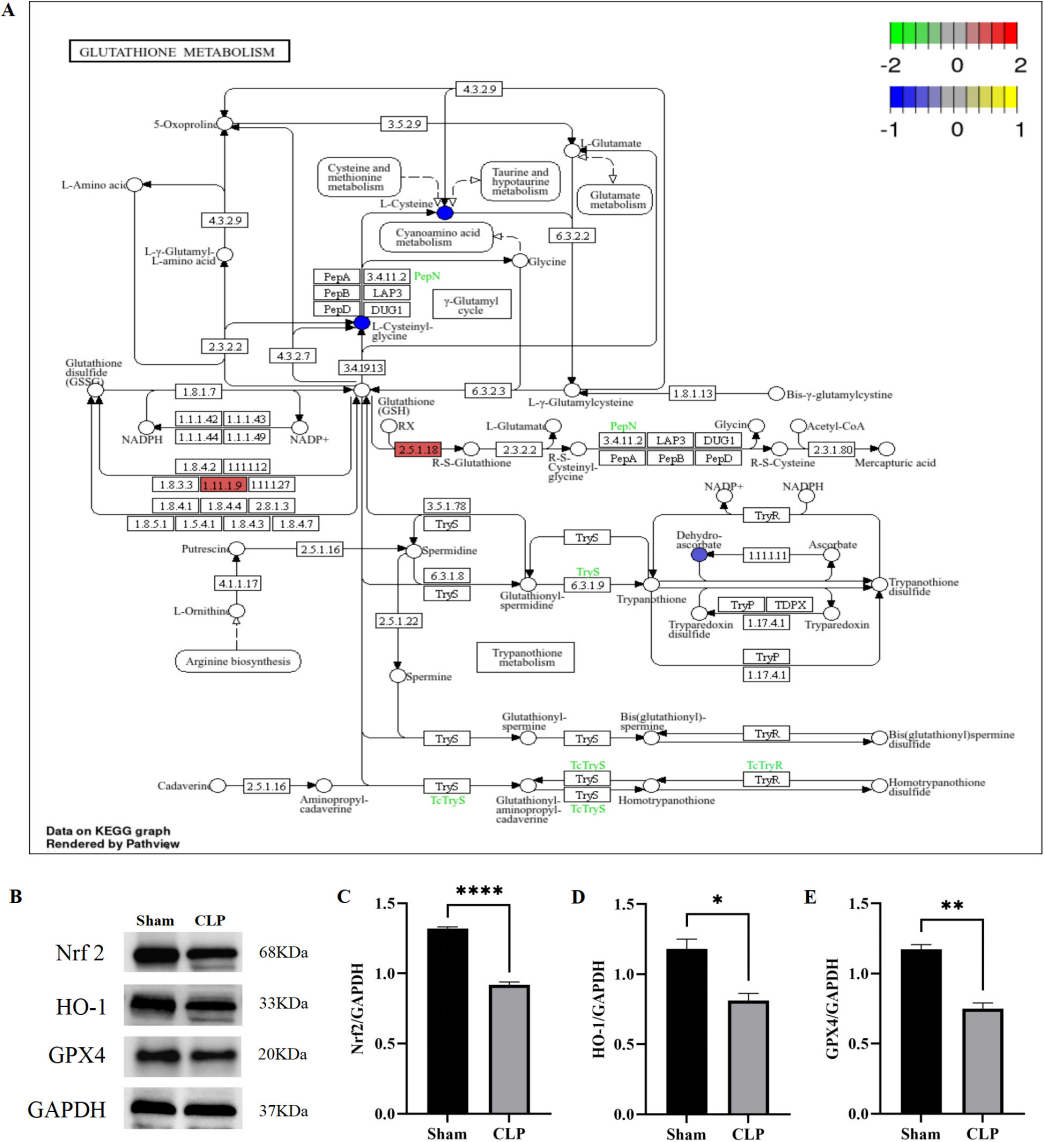
Glutathione metabolism pathway analysis and dysfunction validation in SAE. **(A)** KEGG pathway of glutathione metabolism; **(B–E)**. Representative Western blot images and statistical plots for Nrf2, HO-1, and GPX4. All data are expressed as the mean ± SEM, with each group consisting of three independent biological replicates (*n* = 3). (* *P* < 0.05, ^**^ *P* < 0.01 and ^****^ *P* < 0.0001). Nrf2, nuclear factor erythroid 2-related factor 2; HO-1, heme oxygenase-1; GPX4, peroxidase 4.

Consistent with multi-omics findings, KEGG pathway analysis identified glutathione metabolism as the most significantly perturbed shared pathway (Figure 8A), exhibiting coordinated changes between upregulated antioxidant enzymes such as Gpx3 and Mgst1 alongside depleted glutathione derivatives, supporting redox imbalance as a key pathogenic feature. These integrated analyses linked proteomic and metabolomic data to identify glutathione system dysregulation as a convergent mechanism in SAE. Mechanistic validation via Western blot further confirmed oxidative stress perturbations. Consistent with perturbed glutathione metabolism, CLP mice exhibited significant downregulation of the Nrf2/HO-1 antioxidant axis: reduced Nrf2, a master transcriptional regulator of antioxidant genes, and its target HO-1 were reduced, indicating impaired upstream antioxidant regulation. Concomitantly, GPX4, a glutathione-dependent peroxidase critical for lipid peroxide clearance—was downregulated in CLP mice, reflecting compromised downstream antioxidant function (Figures 8B–E, *P* < 0.05).

The aforementioned abnormalities at the molecular level (downregulation of the Nrf2/HO-1 axis and GPX4) and subcellular structural level (neuronal somata and mitochondrial impairment) were consistent with the GSH metabolism perturbations identified by multi-omics analyses, collectively reflecting oxidative stress-associated damage in the hippocampus during SAE.

## 4 Discussion

The present study elucidates the critical role of glutathione metabolism in mediating oxidative hippocampal injury in mid-late SAE. Through integrated proteomic and metabolomic analyses, we identified a “high-inflammation, high-oxidation, low-metabolism” triad in the hippocampus of CLP-induced SAE mice, providing novel insights into mechanisms underlying persistent cognitive deficits in SAE survivors.

### 4.1 Clinical relevance of the CLP model

The CLP model was selected for its unique ability to recapitulate core clinical features of SAE: polymicrobial sepsis, hippocampal neurodegeneration, and chronic cognitive impairment (Jiang et al., 2023; Yin et al., 2023). Focusing on the subacute-chronic phase (7 days post-CLP) aligns with clinical observations of delayed hippocampal injury and memory decline (Piva et al., 2023), addressing a gap in research that historically overlooked chronic metabolic drivers of long-term deficits. Key phenotypes (hippocampus-dependent deficits, CA1 degeneration, GSH depletion) and EEG abnormalities (elevated δ/θ waves) directly mirror human SAE (Chu et al., 2023; Hwang et al., 2006; Synek, 1988; Young et al., 1992; Yuan et al., 2020), while TNF-α/IL-1β upregulation and IBa1+ microglial activation validate the “systemic-neuroinflammation” cascade critical to clinical pathogenesis, confirmed the central role of the “systemic inflammation-neuroinflammation” cascade in SAE.

### 4.2 Microglia-mediated oxidative neuronal injury

Microglial activation initiates neuronal damage, and a previous study revealed that the microglial inhibitor minocycline reduces neuronal apoptosis (Mein et al., 2023). Building on this, our in vitro data (LPS-activated BV2-conditioned medium inducing HT22 cell death, ROS overproduction, GSH depletion) recapitulates *in vivo* oxidative stress (Hu et al., 2023; Li et al., 2020; Yi et al., 2023). Notably, microglia-mediated neurotoxicity results from multi-factorial synergy, indicating neuronal injury herein is co-mediated by a “cytokine-toxic metabolite” composite (Suzumura et al., 2006; Ye et al., 2013).

Mechanistically, a microglial “inflammation-oxidative stress” feedforward loop perpetuates injury: Activated microglia secrete neurotoxins, impairing neuronal mitochondrial function and antioxidant capacity (such as GPX4), thus increasing ROS leakage. Excess ROS then activates the NLRP3 inflammasome in microglia, promoting maturation and release of IL-1β (Xiao et al., 2021). This loop disrupts hippocampal function, as SAE mice showed open field/Y-maze deficits, which is consistent with GSH depletion-mediated synaptic plasticity impairment (Wu et al., 2023; Shields et al., 2020; Yoshiyama et al., 2007).

### 4.3 GSH metabolism: a central hub in mid-late SAE

The hippocampus, with its high metabolic demands and low antioxidant capacity, is uniquely vulnerable to septic injury (Yuan et al., 2020). Our multi-omics analyses uncover GSH metabolism as the most disrupted pathway in mid-late SAE—an unprecedented finding. A critical paradox emerged: upregulated antioxidants (Gpx3, Mgst1) could not reverse GSH depletion, signaling exhausted compensation. Metabolomics confirmed this via reduced GSH recycling intermediates (S-lactoylglutathione, cysteine-GSH disulfide) (Kalapos et al., 2022) and energy deficits (depleted carnitines, nicotinamide riboside), directly amplifying ROS (Kalapos et al., 2022).

A Stat1-Gpx3 hub was identified, where TNF-α/IL-1β-induced Stat1 activation suppresses Nrf2 (confirmed by reduced hippocampal Nrf2). This aligns with Sun et al. (2022), who showed Nrf2 regulates GSH synthesis via HO-1/GPX4, and our findings extend this: chronic Nrf2 suppression in mid-late SAE disrupts GSH homeostasis (impairing synthesis/recycling) and diminishes GPX4-mediated anti-lipid peroxidation, promoting ferroptosis.

Kyoto Encyclopedia of Genes and Genomes enrichment linked GSH metabolism to ferroptosis and TNF signaling (Liang et al., 2022; Manabe and Heneka, 2022), reinforcing inflammation-oxidation crosstalk. TNF-α-driven Stat1 activation amplifies neuroinflammation while exacerbating oxidative stress damage, creating a vicious cycle: Inflammation drives oxidative stress, exacerbating neuronal damage and further activating microglia.

### 4.4 Future therapeutic targets

The Nrf2/HO-1/GPX4 axis and GSH pathways are promising therapeutic targets for SAE. Our CLP model showed chronic Nrf2/HO-1/GPX4 suppression (day 7) linked to GSH depletion, ferroptosis, and irreversible antioxidant collapse (Kim and Keum, 2016; Xie et al., 2020), supporting Nrf2 activators (e.g., sulforaphane) or GSH precursors (e.g., NAC) as interventions (Chen et al., 2024; Li et al., 2022; Yusri et al., 2025). Our metabolomics identified reduced nicotinamide riboside (key NAD^+^ precursor) in SAE hippocampi. Its supplementation elevates NAD^+^, activating SIRT1 to regulate metabolism and suppress neuroinflammation (Wang et al., 2021). Collectively, these targets form a multi-target framework addressing SAE’s “inflammation-oxidation-energy failure” core, providing insights for neuroprotection. Translational studies will validate efficacy for precision therapies.

### 4.5 Limitations and future directions

Limitations of the present study include incomplete recapitulation of human SAE heterogeneity by CLP and the lack of Nrf2 knockout/GSH inhibitor validation. Future work will use longitudinal profiling and single-cell sequencing to assess cell-type-specific GSH roles

## 5 Conclusion

**FIGURE 9:**
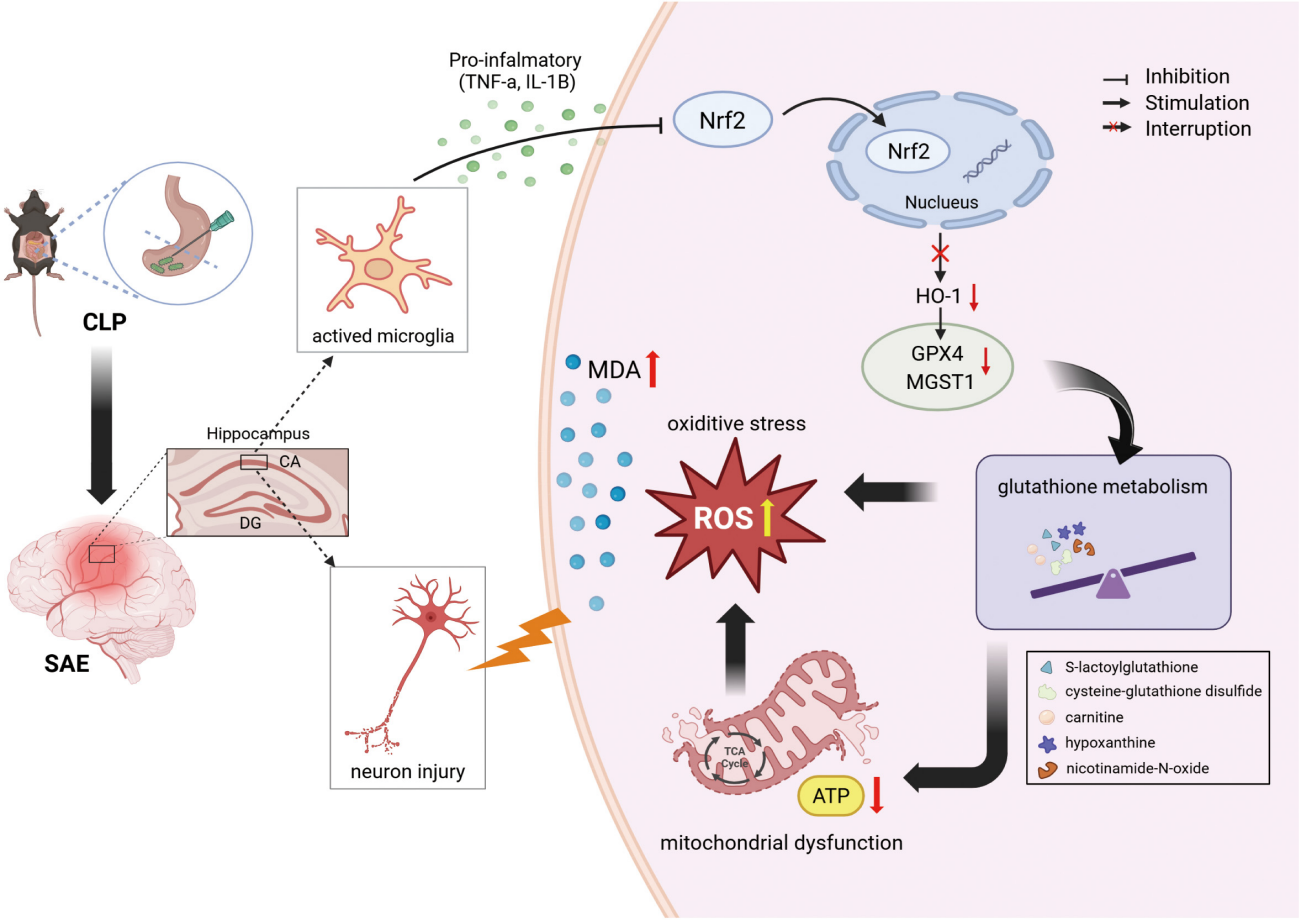
Schematic illustrating the cascade mechanism of cecal ligation and puncture (CLP)-induced hippocampal neuronal damage. Systemic inflammation activates microglia, suppresses the Nrf2/HO-1/GPX4 pathway, and triggers mitochondrial dysfunction, oxidative stress, and glutathione/energy metabolism. Created in https://BioRender.com.

In conclusion, by leveraging multi-omics integration, the present study identified GSH metabolism as a pivotal node in SAE, while providing a comprehensive molecular framework for SAE pathogenesis, and linking microglia-driven neuroinflammation to hippocampal oxidative damage. Specifically, CLP-induced systemic inflammation may activate microglia to secrete pro-inflammatory mediators, which suppress the Nrf2/HO-1/GPX4 redox axis, triggering mitochondrial dysfunction and oxidative stress. Concomitantly, dysregulated GSH metabolism and impaired energy metabolism synergistically exacerbate hippocampal neuronal injury, forming a self-amplifying pathological loop (Figure 9). Future studies should validate these targets in preclinical models and explore their translational potential to develop interventions improving the outcomes of patients with SAE.

## Data Availability

The original contributions presented in this study are included in this article/Supplementary material, further inquiries can be directed to the corresponding author.

## Footnotes

1
https://davidbioinformatics.nih.gov/

2
https://cn.string-db.org/
