# Supplementary material for: Glutathione metabolism as a key regulator of oxidative hippocampal injury in sepsis-associated encephalopathy: an integrated proteomics and metabolomics study
Source: Front Neurosci. 2026 Jan 13;19:1671955. doi: 10.3389/fnins.2025.1671955 (PMC12835233; doi:10.3389/fnins.2025.1671955)
Supplement: Supplementary file 6 [file Data_Sheet_1.docx]

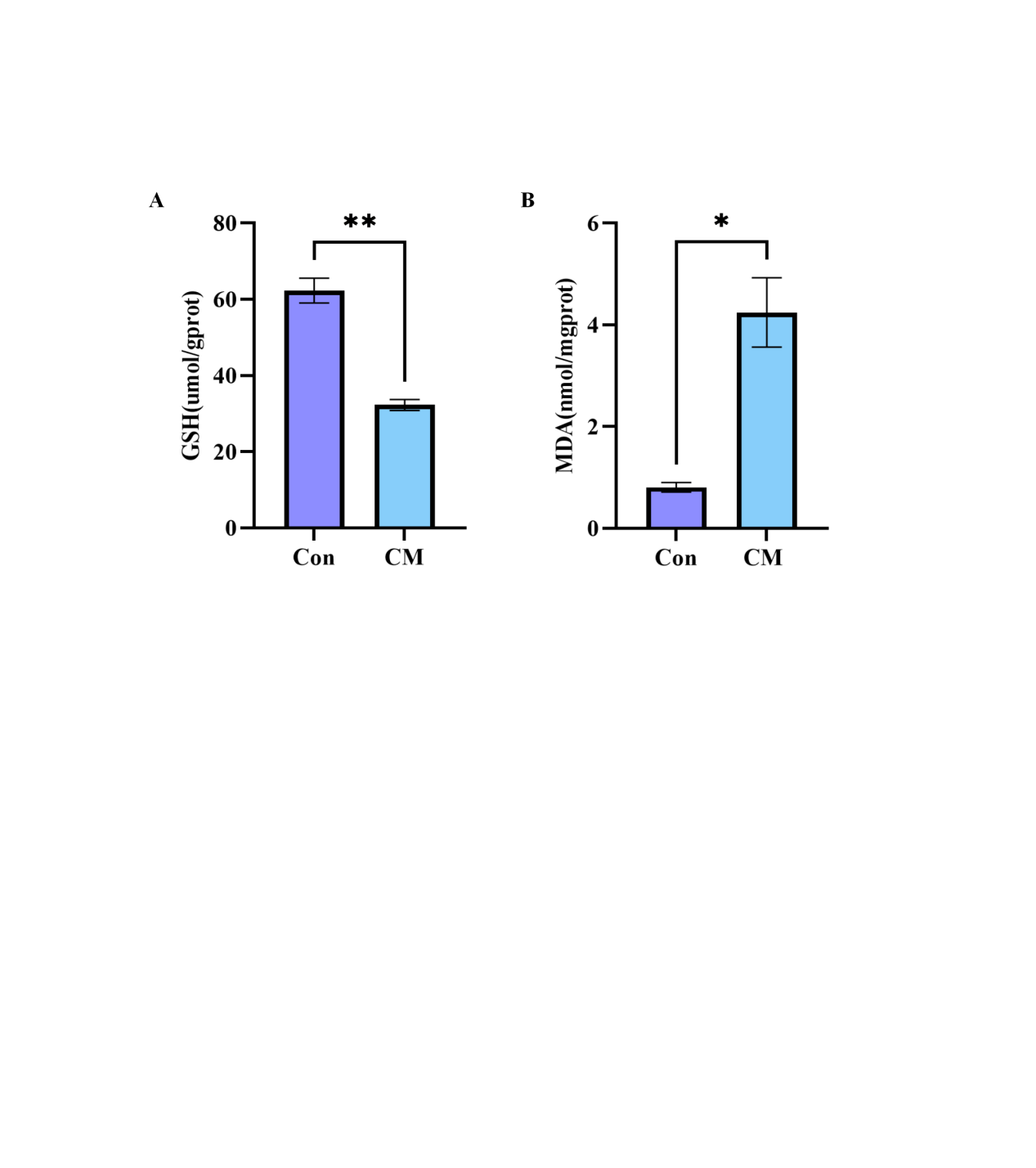


**Supplementary Figure 1. Oxidative stress markers in CM-treated HT22 neurons.** Intracellular glutathione (GSH) levels (A) and malondialdehyde (MDA) content (B) in HT22 neurons treated with conditioned medium (CM) from LPS-activated BV2 microglia. Data are expressed as the mean ± SEM (N = 3 independent experiments). (**p* < 0.05, ***p* < 0.01). Results show concurrent GSH depletion and MDA accumulation in CM-treated HT22 neurons, indicating robust oxidative stress induction.
