# Supplementary material for: Glutathione metabolism as a key regulator of oxidative hippocampal injury in sepsis-associated encephalopathy: an integrated proteomics and metabolomics study
Source: Front Neurosci. 2026 Jan 13;19:1671955. doi: 10.3389/fnins.2025.1671955 (PMC12835233; doi:10.3389/fnins.2025.1671955)
Supplement: Supplementary file 7 [file Data_Sheet_2.docx]

**
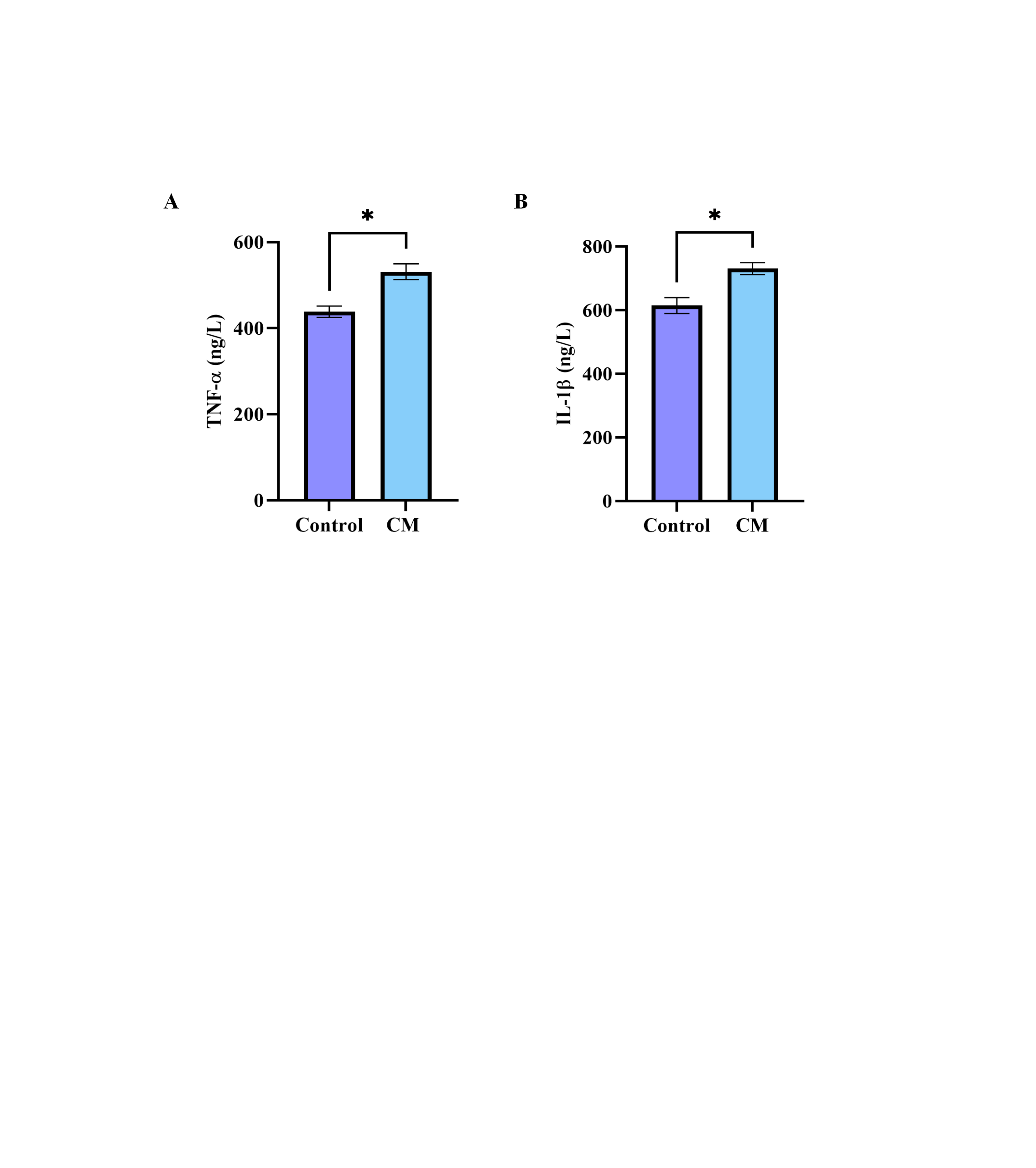
**

**Supplementary Figure 2. Pro-inflammatory cytokine levels in microglia-derived conditioned medium.** Quantification of pro-inflammatory cytokines TNF-α (A) and IL-1β (B) in conditioned medium (CM) from LPS-activated BV2 microglia, measured by ELISA. Data are expressed as the mean ± SEM (N = 3 independent experiments). (*p < 0.05, **p < 0.01). Results show significant upregulation of TNF-α and IL-1β in CM from LPS-activated microglia, confirming enrichment of pro-inflammatory mediators in the conditioned medium.
